# Post-renal Transplantation de novo Renal Cell Carcinoma in a Middle-aged Man

**Published:** 2016-02-01

**Authors:** V. K. Pandya, H. C. Sutariya

**Affiliations:** Department of Radiodiagnosis, IKDRC-ITS, Civil Hospital Campus, Asarwa, Ahmadabad, India

**Keywords:** Allografts, Transplantation, kidney, Carcinoma, renal cell, Kidney diseases, Analgesics, Hematuria, Abdominal pain

## Abstract

Renal cell carcinoma is usually seen in the native kidney but may be seen in the renal allograft. We report a rare case of renal cell carcinoma in a 56-year-old renal allograft recipient who was transplanted for end-stage renal disease induced by analgesic nephropathy. This complication developed after 13 years of renal transplantation. Patient was investigated for hematuria and abdominal pain with a normal renal function. Computed tomography depicted a mass sized 9.0×7.3×6.8 cm that involved the upper pole of the transplant. There was no metastasis. The patient underwent radical allograft nephrectomy for the carcinoma that had extended up to the renal hilum. Histopathological examination revealed Furhman grade-1, clear cell variant, stage pT2 N0 M0. In the last visit, the patient was on maintenance hemodialysis via arterio-venous fistula and planned for cadaveric renal transplantation. Computed tomography could facilitate early diagnosis and proper management of patients with post-renal allograft renal cell carcinoma.

## INTRODUCTION

Renal cell carcinoma (RCC) is more likely to occur in a native kidney of a transplant recipient than in the general population. On the other hand, RCC in a kidney allograft is rare [1]. Primary RCC represents 4.6% of all cancers in transplant recipients and 10% of cancers in kidney grafts [2]. The management of RCC in renal allograft has not yet been established. Renal allograft tumors can be carcinomas that are transmitted by donors, metastatic carcinomas from the recipient’s native organs, or *de novo* carcinomas that occur after transplant. Identification of the origin of a renal allograft tumor can improve therapeutic safety and certainty. Here, we report on the successful diagnosis and treatment of a *de novo* RCC in a renal allograft 13 years after transplantation. 

## CASE REPORT

A 56-year-old man presented with vague abdominal pain, fullness in the right iliac fossa, and gross as well as microscopic hematuria for 20 days. His past medical history revealed he had undergone an uneventful renal transplantation 13 years back in 2001, received from his brother at the age of 42 years for end-stage renal disease induced by analgesic nephropathy. The post-transplantation course was uneventful. The patient maintained stable renal allograft function with serum creatinine around 1 mg/dL and had no rejection episodes. Initially, every six months and later every year periodic ultrasonography and doppler imaging were conducted that had been unremarkable during these 13 years. 

**Figure 1:**
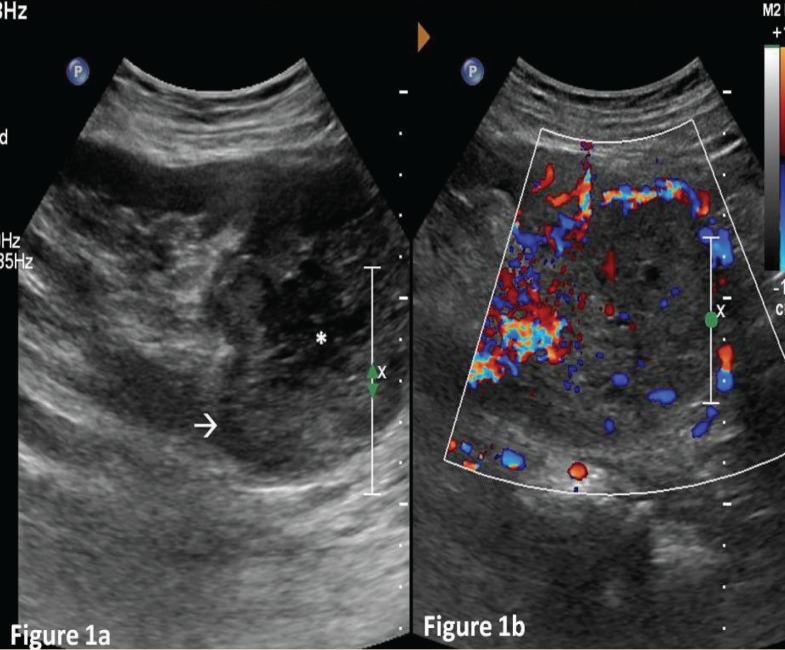
A 56-year-old man with renal cell carcinoma in renal allograft. Findings: Gray scale ultrasound image of renal allograft (a) heterogeneous mass lesion at the upper pole of renal allograft (white arrow) with internal hypoechoic region (asterisk) suggesting necrosis; (b) the mass lesion showed internal as well as peripheral vascularity on color doppler study (Technique: 2D-Ultrasound image scanned with Phillips IU-22 scanner and curvilinear C5-1 probe with frequency

**Figure 2:**
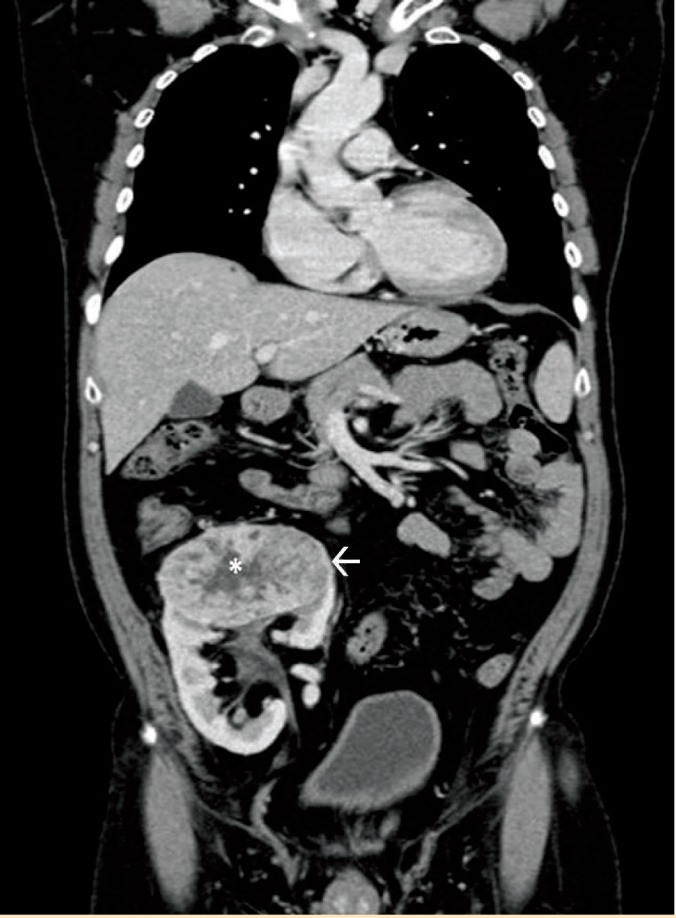
A 56-year-old man with renal cell carcinoma in renal allograft. Findings: Coronal CT with intravenous contrast, arterial phase of abdomen showing heterogeneously enhancing mass lesion at the upper pole of renal allograft (white arrow) in the right iliac fossa with internal hypodense nonenhancing region (asterisk) suggesting necrosis (Technique: Siemens Somatom sensation 64-slice CT scanner, Coronal CT, KV 120, Eff mAs 105, Slice thickness 5 mm. Contrast: Iohexol 350, 70 mL, Arterial Phase

**Figure 3:**
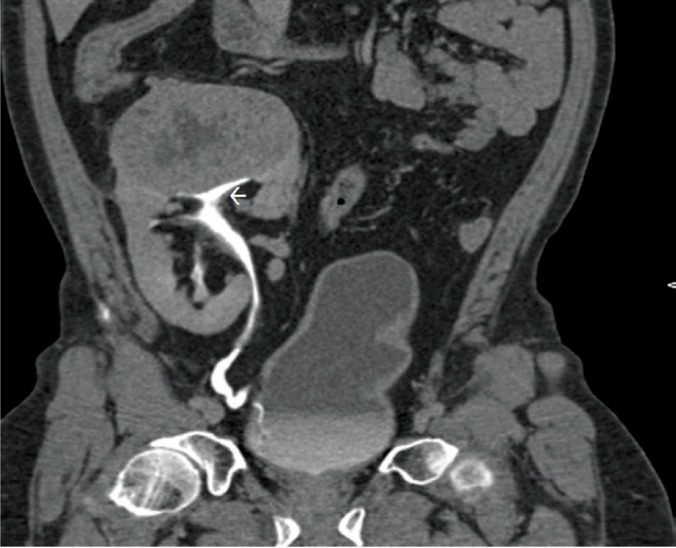
A 56-year-old man with renal cell carcinoma in renal allograft. Findings: Coronal CT of the abdomen with intravenous contrast. Curved MPR reconstruction of delayed image showing normal contrast excretion in the renal allograft with heterogeneously enhancing mass lesion at the upper pole of renal allograft involving PC system in the upper pole renal allograft (white arrow) (Technique: Siemens Somatom sensation 64-slice CT scanner, Coronal CT, KV 120, Eff mAs 105, Slice thickness 5 mm. Contrast: Iohexol 350, 70 mL, Delayed Phase [10 min

On evaluation with ultrasound, a heterogeneous vascular mass sized 9.0×7.3×6.8 cm was seen involving the upper pole of the renal allograft (Fig 1). Doppler study of the graft vessels showed no abnormality. Contrast enhanced computed tomography (CT) showed heterogeneously enhancing mass lesion involving the upper pole with surrounding neo-vascularity (Fig 2) with no evidence of internal calcification or fat density. The mass lesion showed internal necrotic areas and involved the pelvicalyceal system in the upper pole (Fig 3). No evidence of local or distant metastasis was seen. The renal allograft showed normal parenchymal enhancement and prompt contrast excretion. The transplanted vessels appeared normal in terms of course and caliber. The radiological features indicated probable diagnosis of locally confined malignant mass lesion involving the upper pole of the renal allograft.

**Figure 4:**
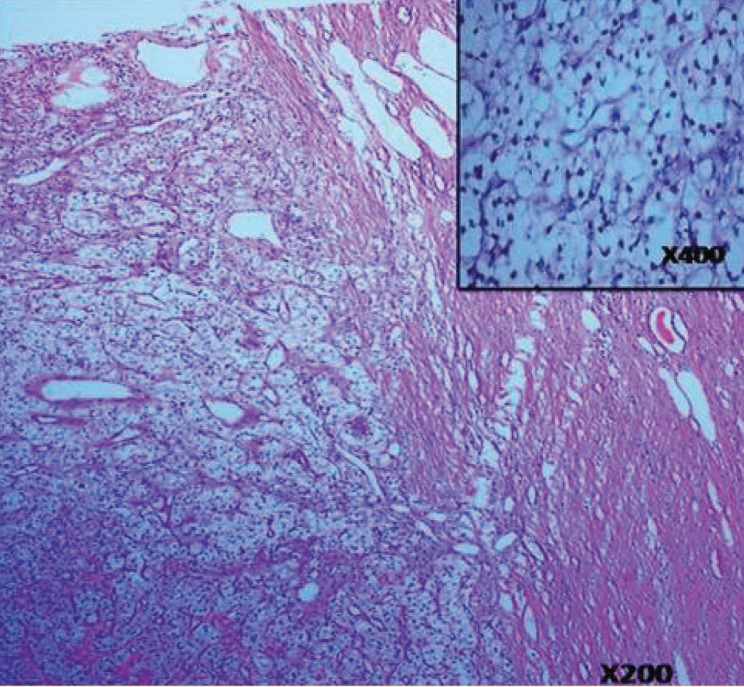
A 56-year-old man with renal cell carcinoma in renal allograft. Findings: Tumor with capsule. Tumor cells are arranged in organoid pattern/fascicles. Individual cells (inset) are round to polygonal, moderately large and having clear or rarely fine granular cytoplasm with well-defined borders and round vesicular nuclei with mild anisonucleosis with rarely prominent nucleoli (H&E, ×200 and ×400 [inset

The patient underwent radical graft nephrectomy. On table, the mass was large and found to extend up to the hilum of the transplant. No local metastasis was found intra-operatively. Histopathology confirmed the diagnosis of renal cell carcinoma, clear cell variant, Furhman grade-1, stage pT2 N0 M0 (Fig 4). 

On follow-up, two months after the graft nephrectomy, his serum creatinine was 4 mg/dL and he was enrolled in the waiting list for cadaveric renal transplantation.

## DISCUSSION

Necessity of immunosuppressants poses a greater risk of developing *de novo* malignancies in post-transplantation patients. RCC occurs twice as frequently as in patients who have received a transplant. RCC in renal allograft can be present either in the donor, as a pre-existent neoplasm in the recipient prior to transplantation, as *de novo* malignancy arising post-transplantation in the native recipient kidneys, or in the graft. Patients with end-stage renal disease have additional risk of developing renal malignancy. 

The overall incidence of malignancy after renal transplantation is 3–5 times higher than in general population [3]. Non-melanoma skin cancer and lymphoma are the most common malignancies followed by RCC [4]. The incidence of RCC is 0.5% to 1.5% among renal transplant patients occurring in the native kidneys or allograft [5, 6]. 

Only few cases of RCC in renal allograft have been reported so far. In two large series published, RCC was observed in 0.16% of grafts of 1250 renal allograft recipients in one series [7], and 0.47% out of 1073 patients in another series [8]. These tumors are reported to have appeared from 1 to 213.5 (average 52) months after transplantation [9]. The length of time a patient remains on dialysis increases risk of RCC in native kidneys with a reported cancer incidence of 1.6%–4.2% [10, 11]. RCC of kidney grafts is supposedly less aggressive than that of native kidneys [6]. There are geographic variations in RCC incidence. A relatively higher incidence of RCC is found in Canada, where in screening of 1000 asymptomatic business executives, four were diagnosed with RCC (0.4%) [12].

The first case of an allograft RCC was published in 1976 [13]. Radical nephrectomy was the preferred approach for oncological control with subsequent graft loss and long-term hemodialysis [14-16]. Dialysis is associated with poor survival compared to RCC. Patients on hemodialysis have a reported overall 5-year survival rate of 34% compared to a 70%–90% and 50%–70% five-year survival rate for patients with stage I and stage II RCC, respectively [17]. Poorer survival outcomes for dialysis and improved experience with radical nephrectomy prompted review of transplant nephrectomy as the standard of care in these patients.

Majority of the renal allograft recipients are asymptomatic due to absence of the graft innervation. Therefore, RCC is found incidentally in most cases during periodic follow-up. However, rarely RCC in a renal allograft may present with abdominal pain and hematuria, as was observed in our patient. If it has distant metastasis, organ specific complaints may be present.

The superficial position of the transplanted kidney allows the use of high-frequency transducers, which improves the signal-to-noise ratio and the spatial resolution, and makes the analysis of the tumor content more accurate. Doppler technique facilitates the visualization of the vascularity of the lesion, with large peripheral vessels and central tumor vessels. On ultrasound, the RCC in renal allograft appears as a heterogeneous mass lesion with internal vascularity on color doppler study. Internal necrotic areas may be seen as anechoic regions. On CT, the lesion appears as heterogeneously enhancing mass lesion with internal hyper-dense hemorrhage or non-enhancing hypodense necrosis. Presence of internal calcification foci or fat density can be seen on CT. CT can also precisely detect any metastatic lesions.

The diagnosis is made by ultrasonography; CT can detect the extension of the mass with local and distant spread and thus guide better management. Treatment of these tumors is usually consisted of graft removal, modification of immunosuppression, and return to hemodialysis, with obvious psychologic repercussions for the patient. Tumors less than 4 cm and in peripheral location can be managed with nephron sparing surgery where the loss of whole graft is prevented and patient can continue with the tumor-free part of a functioning renal allograft. Unfortunately, in our patient it was not possible to perform nephron sparing surgery due to large tumor size. Therefore, radical graft nephrectomy was carried out.

The lesion in our patient had the classic ultrasound and CT features described for RCC. Other entities, which should be considered for differential diagnosis included renal oncocytoma, chromophobe RCC, angiomyolipoma, transitional cell carcinoma, lymphoproliferative disease, infection, and hemorrhagic cyst or metastasis. However, in the present case, the lesion in the renal allograft did not have any evidence of central scar or fat density; neither was it in favor of any lymphoproliferative disorders, infectious diseases, or metastasis. Therefore, we directly arrived at the probable diagnosis of RCC in the renal allograft, which was later confirmed by histopathology.

In summary, periodic evaluation of the renal allograft is very useful to detect any mass lesion in it. Any suspicious lesion on primary ultrasound imaging should be investigated by CT or MRI to facilitate early diagnosis and proper management including nephron sparing surgery to prevent catastrophe and to preserve graft function. 
